# Effect of M-Learning on promoting the awareness of faculty members of the universities of medical sciences of Iran about their employment regulations in 2020

**DOI:** 10.3389/fpubh.2022.947478

**Published:** 2022-09-02

**Authors:** Abdolreza Gilavand

**Affiliations:** Department of Education Development Center, Ahvaz Jundishapur University of Medical Sciences, Ahvaz, Iran

**Keywords:** M-Learning, App, employment regulations, faculty members, Iran

## Abstract

**Introduction:**

New technologies enable universities to incorporate innovative teaching-learning strategies into their curricula. Therefore, this research investigates the effect of mobile learning on promoting the awareness of faculty members of the universities of medical sciences of Iran about their employment regulations.

**Materials and methods:**

The statistical population of this research included all faculty members of Ahvaz Jundishapur University of Medical Sciences in southwestern Iran. One hundred and fourteen people entered the quasi-experimental study through convenience sampling. First, we sent the designed mobile app to them through social networks to install on their phone. We measured their awareness about the app and M-Learning through a researcher-constructed questionnaire.

**Results:**

M-Learning and App was able to increase the awareness of faculty members, which was significant according to their academic rank and work experience. Faculty members who were professor and instructor, as well as those who had e under 5 years or 11–20 years of experience gained more awareness (*P* = 0.00). It was not significant in other variables. Ninety eight percent of the faculty members considered the technical capabilities of the designed app appropriate. Ninety seven percentage of the faculty members suggested M-Learning for teaching similar subjects in their profession.

**Discussion and conclusion:**

The faculty members had a positive attitude toward this designed educational app and M-Learning. Designing similar mobile training apps can improve their professional performance.

## Introduction

Nowadays, many schools and universities use electronic learning and mobile learning to offer their courses and encourage students to use smart devices for their educational purposes (1). Use of mobile devices and apps installed on them that have high capabilities have increased in recent years in schools and universities for learning purposes (2). The use of M-Learning in medical education is expanding. Some of the most important advantages of M-Learning are possibility of learning at any time, saving time and money, continuous communication between learner and teacher, ability to self-assess while learning, high accessibility, ease for the learner, increase in motivation and diversity in learning (use of audio and video that makes attractive and better learning and memory retention) (3). Even in some medical faculties, M-Learning is officially included in the student curriculum. For example, since 2013, the University of Helsinki has given an iPad for their use to the new medical and dental students (1, 3). New technologies enable universities to incorporate innovative teaching-learning strategies into their curricula. M-Learning has significant potential for the classroom (4). M-learning is a popular field in educational organizations, companies as well as for individual study (5). Studies have shown that M-learning is more effective than traditional learning methods (6, 7). M-Learning is also usable as a complement to traditional learning (8). During the Covid-19 pandemic, due to the closure of universities and educational institutions, the role of M-Learning in filling the educational gap and compensating for face-to-face training was quite evident (5).

There are 68 independent universities or colleges of medical sciences in Iran with more than 20,000 faculty members. The Universities and Colleges provide all their employment regulations (such as employment, retirement, promotion, leave, evaluation, etc.) in a bylaw called the employment regulations of the faculty members of the Ministry of Health and Medical Education (Universities of medical sciences) of Iran. The latest amendment to the employment regulations of the faculty members of universities of medical sciences affiliated to the Ministry of Health and Medical Education of Iran was approved in 2012 and notified all medical universities for implementation. This regulation has been amended many times during the last 10 years in the form of various directives and instructions. However, the Ministry of Health and Medical Education of Iran has not yet provided a new version of it. Only a paper version of it is available presently and there is no designed App for it. Therefore, according to his field of study and work experience in recruiting faculty members of Ahvaz Jundishapur University of Medical Sciences and with full knowledge of all these changes, the researcher has prepared a mobile app based on this bylaw and its subsequent amendments and directives. In the “Appendices” section, he has added several widely used directives and instructions for faculty members. Thus, we conducted this research to examine the M-Learning effect in promoting the awareness of the faculty members of the universities of medical sciences of Iran an about their employment regulations.

## Materials and methods

The statistical population of this research included all faculty members of Ahvaz Jundishapur University of Medical Sciences in southwestern Iran (*N* = 651). One hundred and fourteen people entered the quasi-experimental study through convenience sampling. In this study, first we sent a mobile app designed by the researcher to faculty members through WhatsApp and Telegram social networks to install on their phones. We measured then their awareness about the designed mobile app through M-Learning after installing the software. This App is also available to the public gratuitously through Iranian internet markets, has been downloaded many times and has had a 5-star rating. Due to some of its unique capabilities, the App provides the possibility of learning administrative and employment regulations better and easier, and meets all the information needs of faculty members. It will also always be available to users. Because it is complete and comprehensive, it has the ability to search for content and updates. The data collection tool was a researcher-made questionnaire. It consists of 3 parts. The first part includes demographic information (5 questions). Its second part has 12 questions according to the content of the administrative and employment regulations of the faculty members of the Ministry of Health and Medical Education of Iran (such as employment, retirement, promotion, leave, evaluation, etc.,). The third part has 2 general questions about the form and technical capabilities of this App and the opinion of faculty members regarding the use of M-Learning for teaching similar subjects in their profession. We designed this questionnaire based on a 4-grade Likert scale (very high, high, moderate and low). Leveling of the questionnaire to evaluate the level of awareness: 12–24 is low, 25–36 is medium, 37–48 is high, and 49–60 is very high. This one-year study was conducted in 2020. The validity and reliability of this questionnaire has been confirmable. The experts confirmed the content validity of the questionnaire, so that the questionnaire became available for 10 educational experts. We used re-test to determine the reliability of the tool; so we distributed 20 questionnaires among the faculty members with an interval of 2 weeks and examined the reliability of the questions. Cronbach's alpha by 95% confirmed its reliability. We analyzed the data through SPSS software version 24 at a significant level of 0.05. We have carried out this research in line with the research plan No. 961036 approved by the National Center for Medical Education Researches of the Ministry of Health and Medical Education (NASR). Its ethical code is IR.AJUMS.REC.2018.583.

## Results

Table 1 shows the impact of M-Learning and the designed App in promoting the awareness of faculty members about employment regulations according to their demographic variables. The results of this research showed that the designed App was able to increase their awareness, which was significant according to their academic rank and work experience. Faculty members who had the scientific ranks of professor and instructor gained more awareness (*P* = 0.00). Faculty members who had work experience under 5 years or 11–20 years of experience gained more awareness (*P* = 0.00) but it was not significant in other variables.

**Table 1:** Effect of M-Learning and the designed App in promoting the awareness of faculty members.

**Variable**		**Number**	**Percentage**	**Mean**	**Standard deviation**	***P*-value**
Gender	Man	66	57.9	46.9	10.59	0.514
	Woman	84	41.1	47.68	10.87	
	Total	114				
Academic rank	Instructor	9	7.9	50	4.82	0.00
	Assistant professor	56	49.1	47.48	11.18	
	Associate professor	36	31.6	42.02	9.51	
	Professor	13	11.4	57	6	
	Total	114	100			
Work experience	Under 5 years	18	15.8	52.17	9.22	0.00
	5–10	34	29.8	45.15	7.54	
	11–20	35	30.7	51.63	10.74	
	21–30	19	16.7	38.21	9.79	
	Above 30	8	7.0	44.50	12.64	
	Total	114	100			
Type of service	Clinical science	62	52.48	46.03	11.68	0.273
	Basic science	52	59.13	48.50	9.43	
	Total	114	100			
Type of employment	Permanent	57	58.4	44.86	11.88	0.94
	Contractual employment	35	31.0	49.88	697	
	Service commitment	13	10.6	50.41	11.20	
	Total	114	100			

Table 2 shows the impact of M-Learning and the designed App in promoting the level of faculty members' awareness of employment regulations according to the content of the App. The results showed that M-Learning and the designed App were able to increase the awareness of faculty members about all chapters and their contents. The chapters of “Payment and Benefit System”, “Leaves” and “Basic promotion and the promotion of faculty members” had a higher score respectively.

**Table 2:** Effect of M-Learning in promoting the awareness of faculty members according to the content of the App.

**Row**	**Questions**	**Very high**	**High**	**Moderate**	**Low**
1	How much did it raise your awareness after installing and using this App regarding the chapter “Generalities and definitions of the faculty employment regulations”?	33.3%	34.3%	17.5%	14.9%
2	How much did it raise your awareness after installing and using this App regarding the chapter “Entering the service and employing faculty members”?	30.7%	32.4%	28.1%	8.8%
3	How much did it raise your awareness after installing and using this App regarding the chapter “Scale promotion and promotion of faculty members”?	37.7%	31.6%	23.7%	7.0%
4	How much did raise your awareness after installing and using this App regarding the chapter “Missions of faculty members”?	40.4%	25.4%	26.3%	7.9%
5	What was your level of awareness after installing and using this App regarding the chapter “Study opportunities for faculty members”?	35.1%	34.2%	16.7%	14.0%
6	How much did it raise your awareness after installing and using this app about the chapter “System of payment of salaries and benefits of faculty members”?	36.8%	41.3%	12.3%	9.6%
7	How much did it raise your awareness after installing and using this App regarding the chapter “Assessing the performance of faculty members”?	34.2%	34.2%	19.3%	12.3%
8	How much did it raise your awareness after installing and using this App regarding the chapter “Retirement of faculty members”?	37.7%	28.1%	20.2%	14.0%
9	How much did it raise your awareness after installing and using this App about the chapter “faculty leave”?	39.5%	33.3%	16.7%	10.6%
10	How much did it raise your awareness after installing and using this App about the chapter “General Assignments of Faculty Members”?	36.8%	30.8%	21.9%	10.5%
11	How much did it raise your awareness after installing and using this App regarding the chapter “Other regulations of faculty members”?	32.5%	32.5%	23.7%	11.3%
12	How much did it raise your awareness after installing and using this App about the chapter “Attachments (some widely used circulars and instructions)”?	34.2%	24.5%	28.1%	13.2%

At the end of the questionnaire, we asked 2 general questions about the form and technical capabilities of this App and the opinion of faculty members regarding the use of M-Learning for teaching similar professional subjects. Ninety eight percent of faculty members considered the technical capabilities of the designed App appropriate (such as form and content, categorization of subjects, and its ability of searching contents). Ninety seven percent of the faculty members suggested M-Learning to teach similar subjects in their profession.

**Figure 1:**
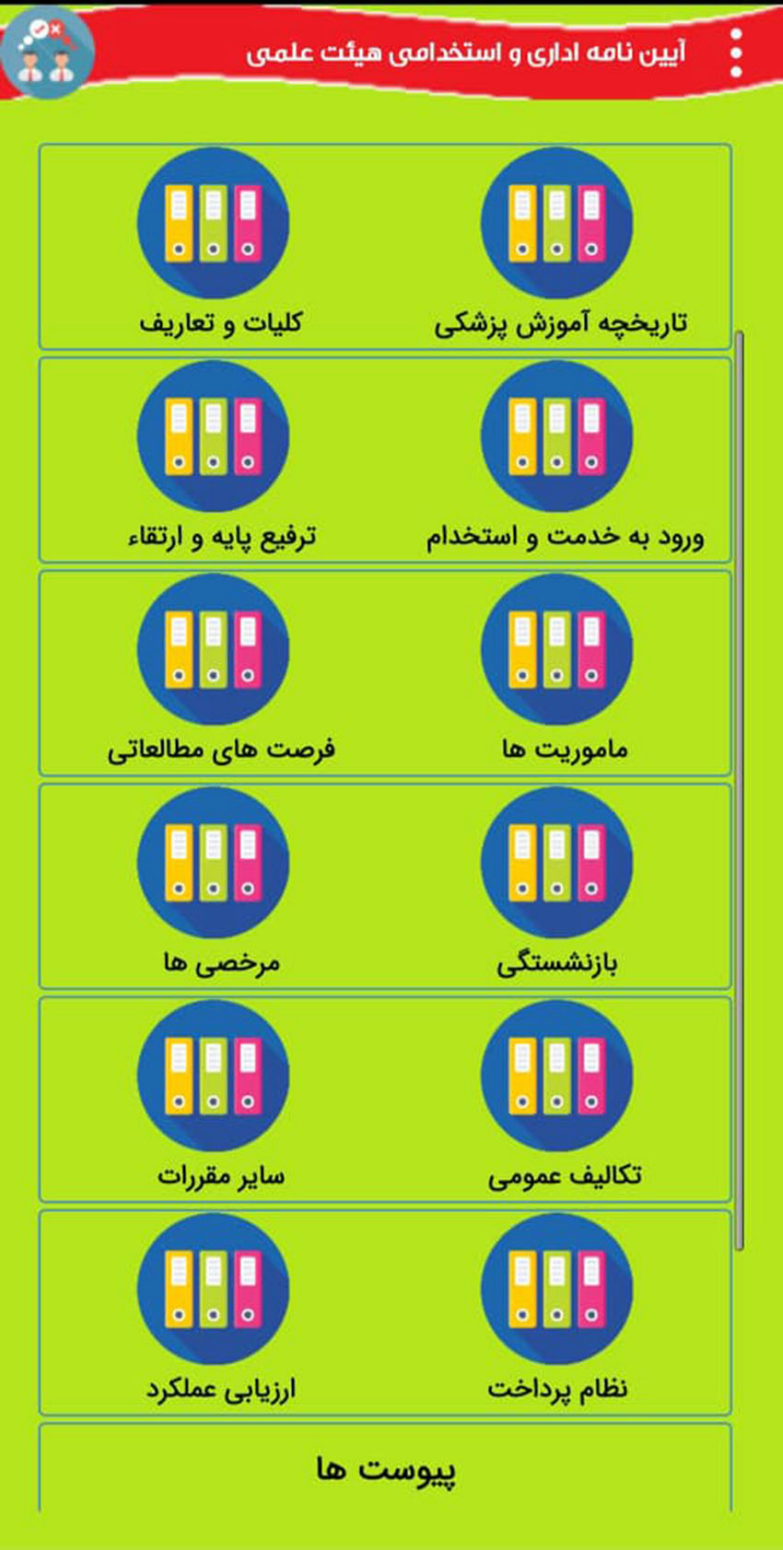
The part of the internal environment of App.

**Figure 2:**
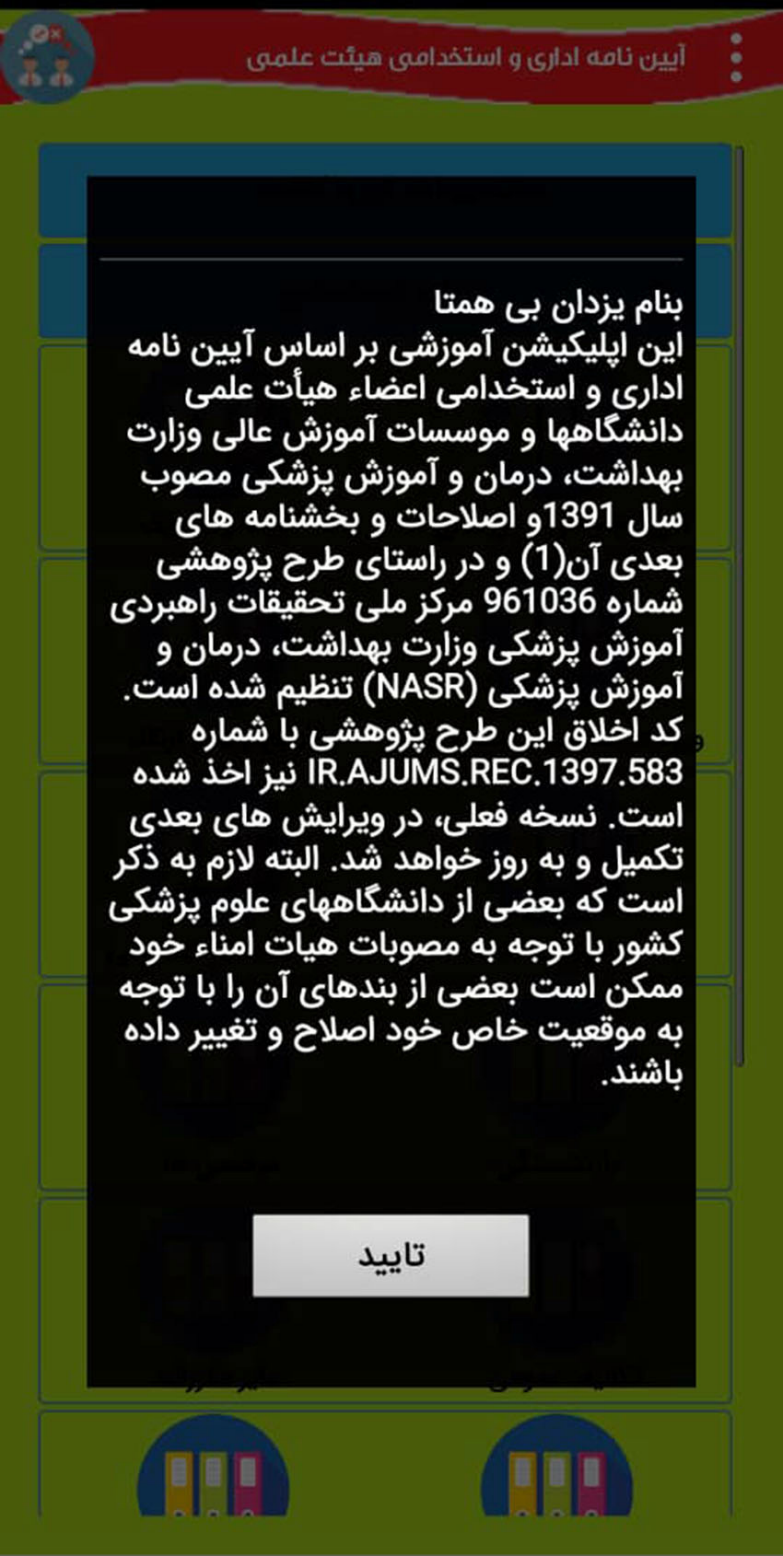
The part of the internal environment of App.

**Figure 3:**
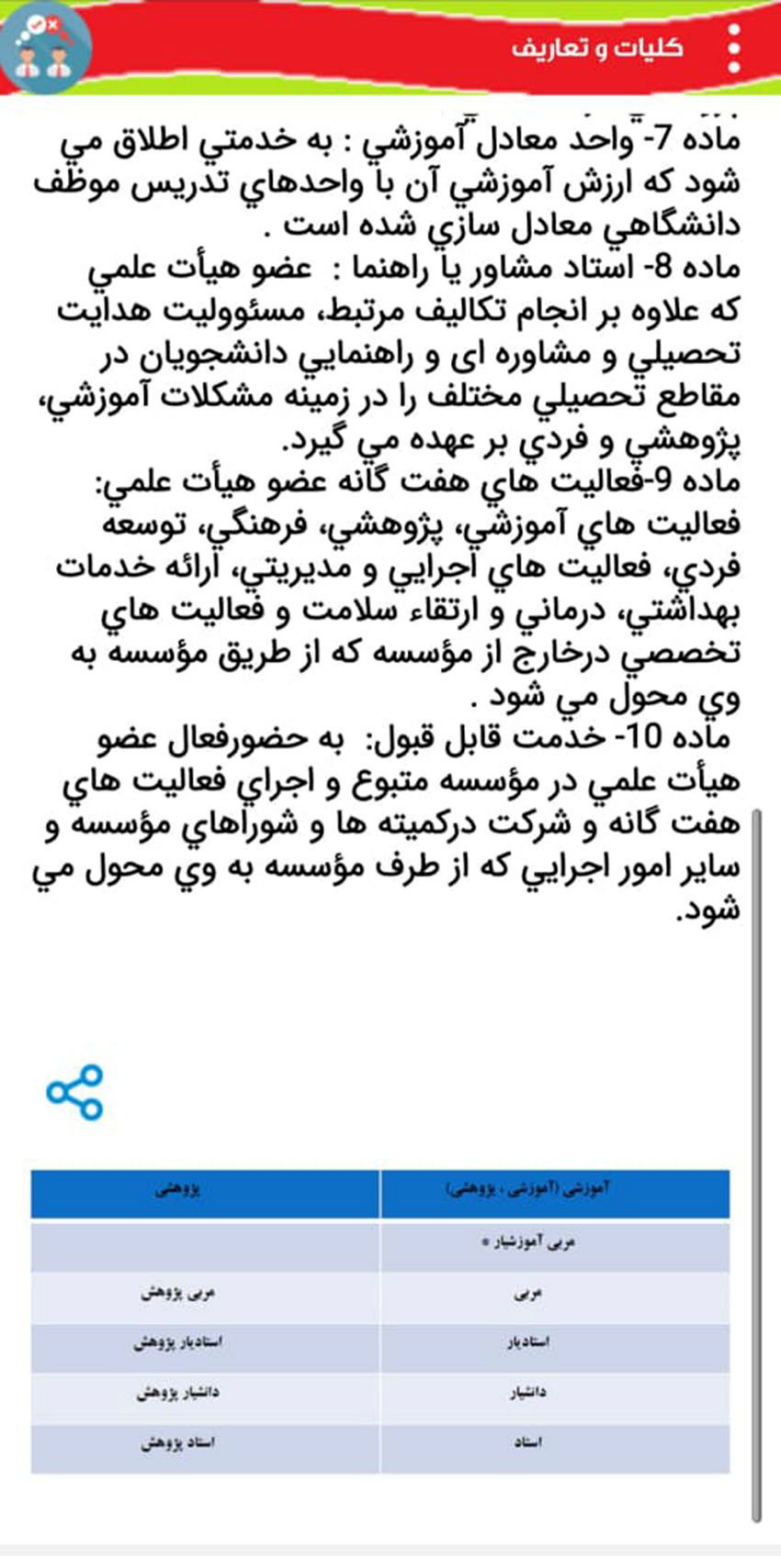
The part of the internal environment of App.

**Figure 4:**
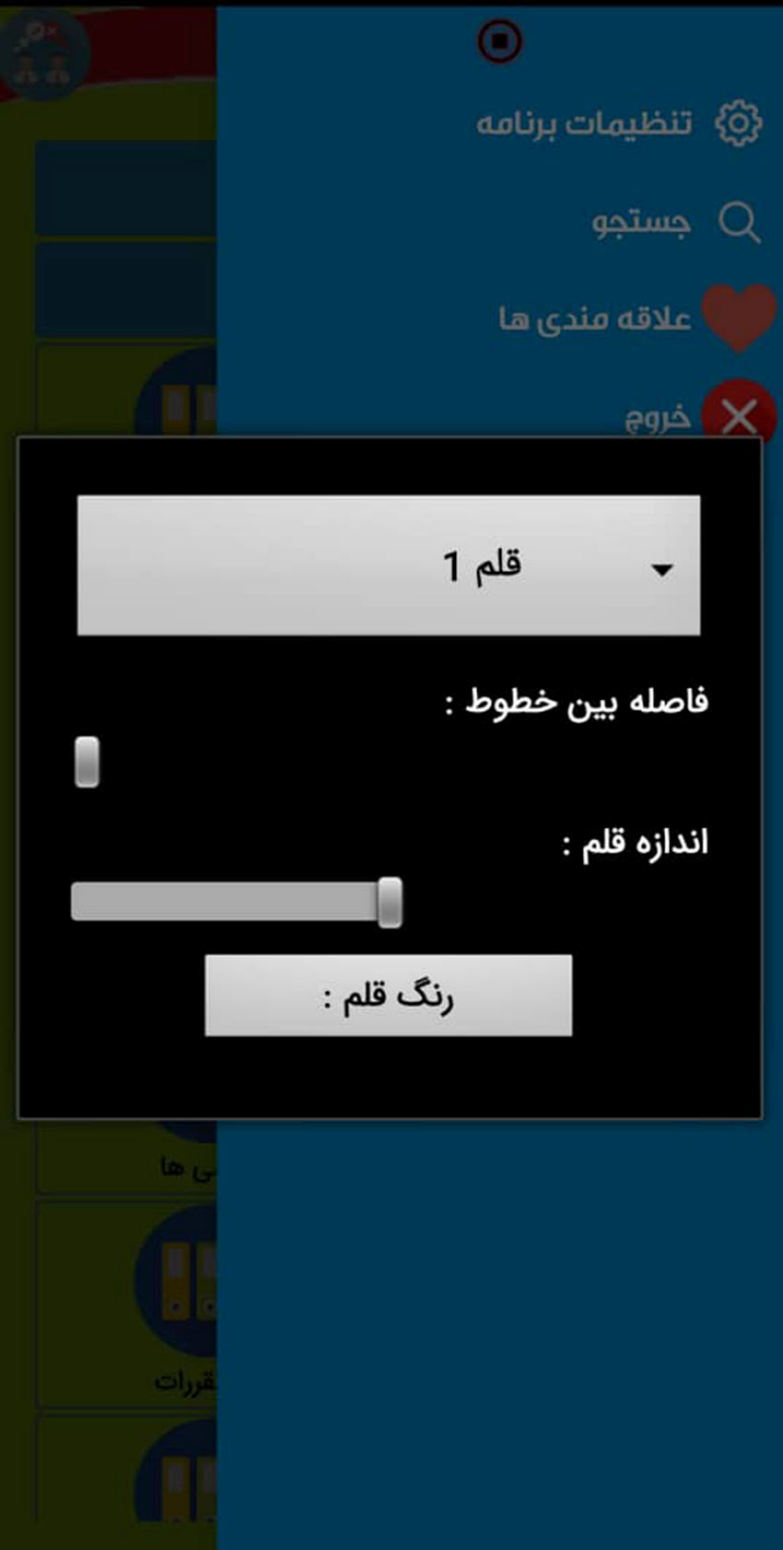
The part of the internal environment of App.

The following Figures 1–4 show part of the internal environment of this app:

## Discussion

The results of this research showed that M-Learning and the designed App, while also obtaining the consent of faculty members, has been able to increase their awareness about the employment regulations. Wyatt et al. conducted a study titled M-learning with participation of students at two Mid-Atlantic universities in the USA. Nursing students and their faculty members participated in the 16-month training course and Personal Digital Assistants (PDAs) were in use in the training activities. The results showed that M-learning is useful in the clinical environment and all students, regardless of learning style, benefit from its use. Students also created a collaborative learning community with each other and with students from other more distant universities (4). In a review, Chen et al. examined nursing students' attitudes toward M-Learning. In this study, they evaluated articles published on the subject until 2021. The results showed that most nursing students were inclined to M-Learning, but the rate of actual use was low (6). In a systematic review, Zhou et al. examined the effectiveness of M-Learning in medical education. In this research, they studied 11 articles published from 2000 to 2014. Seven articles showed that the use of Mobile phones for learning improved effectively their clinical awareness and performance (7). Azizi and Khatony conducted a study on the factors affecting the tendency of medical students to accept M-Learning among students of Iranian medical universities. Eighty three percent of the 332 students who participated in this research welcomed this method (9). The results of a study done by Sharma et al. in India showed that the effectiveness of M-Learning training on nurses' knowledge and attitudes about the prevention and control of MDR tuberculosis was favorable (10). Patil et al. used a mobile app to complement learning and teaching in India, and their research showed that medical students have a positive attitude toward M-learning (11). Keane et al. created a mobile app for medical students called “Urology Med”. During a month from its launch, its users downloaded it 435 times in five countries on three continents and the App gained 5-star rating in the Apple Store (12). Baghcheghi and Koohestani conducted research among medical students in Iran. Their result was that there is a positive and significant relationship between the desire for M-Learning and academic achievement (13). In their study in Spain, Ortega et al. showed that M-learning has played an effective role in improving the learning of nursing students and have stated that teaching activities and methodology in this area need a fundamental revision (14). In their study in Turkey, Suner et al. showed that most dental students saw M-learning useful in dental courses and their attitude toward education through M-learning is positive. They have recommended that the design of educational materials and Apps for mobile devices may increase students' performance (15). In their study in Canada, Darras et al. showed that most medical students prefer to use M-learning to teach radiology over traditional methods (16). The results of the Sitar-Tăut's research in Romania conducted on 311 higher education learners showed that M-learning has been successful during the outbreak of COVID-19. He believes that incorporating gameplay elements into the learning system can be a pleasurable experience to learners (5). In a systematic review article entitled Features, Barriers and Factors Affecting M-learning in Higher Education, Sophonhiranrak et al. reviewed the published articles from 2006 to 2018. They concluded that mobile devices are usable as Learning tools for tasks such as sending homework, reflecting on instant learning experiences, and sharing ideas. At the end of their study, they stated that faculty members should consider three main components (learners and faculty members' readiness, learning management and support systems) in M-learning (17). The results of all this research are consistent with our research.

## Conclusion

The faculty members had a positive attitude toward this designed educational App and M-Learning. Designing similar mobile training Apps can improve their professional performance. We can argue that M-learning has a lot of potential and capabilities. Considering the positive attitudes and perceptions of users on acceptance and use of this technology for educational purposes, it is likely that in the near future it is applied as the first strategy of universities and educational institutions for learning and all e-learning work seamlessly on a mobile device.

## Limitations

We conducted this research based on a self-report survey to evaluate the views of faculty members of a large university of medical sciences in Iran on the effect of using an App designed to promote their awareness of faculty rules and regulations. In regard to other variables, the researcher has ignored them inadvertently and they may be of effect on this assessment.

## Data availability statement

The original contributions presented in the study are included in the article/supplementary material, further inquiries can be directed to the corresponding author.

## Ethics statement

This research was derived from a research project (Ethics Code of IR.AJUMS.REC.2018.583). This paper is a systematic scoping study that relied strictly on the review of existing literature, no human participants were involved. Therefore, ethical approval and consent to participate by human participants was not applicable. Ethical issues (Including plagiarism, misconduct, data fabrication and/or falsification, double publication and/or submission, redundancy, etc.) have been completely observed by the authors.

## Author contributions

AG designed research, conducted research, analyzed data, wrote manuscript, and he had primary responsibility for final content. Author read and approved the final manuscript.

## Funding

This project was funded by the National Agency for Strategic Research in Medical Education, Tehran, Iran (Grant No. 961036).

## Conflict of interest

The author declares that the research was conducted in the absence of any commercial or financial relationships that could be construed as a potential conflict of interest.

## Publisher's note

All claims expressed in this article are solely those of the authors and do not necessarily represent those of their affiliated organizations, or those of the publisher, the editors and the reviewers. Any product that may be evaluated in this article, or claim that may be made by its manufacturer, is not guaranteed or endorsed by the publisher.

## References

[1] Pyörälä E Mäenpää S Heinonen L Folger D Masalin T Hervonen H. The art of note taking with mobile devices in medical education. BMC Med Educ. (2019) 19:96. 10.1186/s12909-019-1529-7 30940152 PMC6446288

[2] Kucuk S Baydas Onlu O Kapakin S. A model for medical students' behavioral intention to use mobile learning. J Med Educ Curric Dev. (2020) 7:2382120520973222. 10.1177/2382120520973222 33313399 PMC7716062

[3] Walsh K. Mobile learning in medical education: review. Ethiop J Health Sci. (2015) 25:363–6. 10.4314/ejhs.v25i4.10 26949301 PMC4762975

[4] Wyatt TH Krauskopf PB Gaylord NM Ward A Huffstutler-Hawkins S Goodwin L. Cooperative m-learning with nurse practitioner students. Nurs Educ Perspect. (2010) 31:109–13. 20455369

[5] Sitar-Tăut DA. Mobile learning acceptance in social distancing during the COVID-19 outbreak: The mediation effect of hedonic motivation. Hum Behav Emerg Technol. (2021) 24:10.1002/hbe2.261. 10.1002/hbe2.261 34222833 PMC8239841

[6] Chen B Yang T Wang Y Xiao L Xu C Shen Y Qin Q Wang Y Li C Chen F Leng Y Pu Y Sun Z. Nursing students' attitudes toward mobile learning: an integrative review. Int J Nurs Sci. (2021) 8:477–85. 10.1016/j.ijnss.2021.08.004 34631998 PMC8488805

[7] Zhou Y Yang Y Liu L Zeng Z. [Effectiveness of mobile learning in medical education: a systematic review]. Nan Fang Yi Ke Da Xue Xue Bao. (2018) 38:1395–400. Chinese. 10.12122/j.issn.1673-4254.2018.11.20 30514692

[8] Klímová B. Mobile Learning in Medical Education. J Med Syst. 2018; 12;42(10):194. 10.1007/s10916-018-1056-9 30209627

[9] Azizi SM Khatony A. Investigating factors affecting on medical sciences students' intention to adopt mobile learning. BMC Med Educ. (2019) 19:381. 10.1186/s12909-019-1831-4 31638977 PMC6802341

[10] Sharma SK Mandal A Mishra M. Effectiveness of m-learning on knowledge and attitude of nurses about the prevention and control of MDR TB: a quasi-randomized study. Indian J Tuberc. (2021) 68:3–8. 10.1016/j.ijtb.2020.10.013 33641848

[11] Patil RN Almale BD Patil M Gujrathi A Dhakne-Palwe S Patil AR. Attitudes and perceptions of medical undergraduates towards mobile learning (M-learning). J Clin Diagn Res. (2016) 10:JC06–10. 10.7860/JCDR/2016/20214.8682 27891356 PMC5121694

[12] Keane KG Bhatt NR Collins PM Flynn RJ Manecksha RP. Urology at your fingertips: the development of a urology m-learning app for medical students. Transl Androl Urol. (2021) 10:1152–9. 10.21037/tau-20-1245 33850750 PMC8039588

[13] Baghcheghi N Koohestani HR. The relationship between the willingness to mobile learning and educational achievements in health-care professional students. J Educ Health Promot. (2021) 10:378. 10.4103/jehp.jehp_1491_20 34912914 PMC8641724

[14] Ortega Lde M Plata RB Jiménez Rodríguez ML Hilera González JR Martínez Herráiz JJ Gutiérrez De Mesa JA. Using M-learning on nursing courses to improve learning. Comput Inform Nurs. (2011) 29:TC98–104. 10.1097/NCN.0b013e3182285d2c 21701278

[15] Suner A Yilmaz Y Pişkin B. Mobile learning in dentistry: usage habits, attitudes and perceptions of undergraduate students. PeerJ. (2019) 7:e7391. 10.7717/peerj.7391 31392099 PMC6673424

[16] Darras KE G van Merriënboer JJ Toom M Roberson ND H de Bruin AB Nicolaou S Forster BB. Developing the evidence base for M-learning in undergraduate radiology education: identifying learner preferences for mobile apps. Can Assoc Radiol J. (2019) 70:320–6. 10.1016/j.carj.2019.03.007 31300315

[17] Sophonhiranrak S. Features, barriers, and influencing factors of mobile learning in higher education: a systematic review. Heliyon. (2021) 7:e06696. 10.1016/j.heliyon.2021.e06696 33869873 PMC8045005

